# Unveiling biological activities of biosynthesized starch/silver-selenium nanocomposite using *Cladosporium cladosporioides* CBS 174.62

**DOI:** 10.1186/s12866-024-03228-1

**Published:** 2024-03-08

**Authors:** Fathy M. Elkady, Amr H. Hashem, Salem S. Salem, Gharieb S. El-Sayyad, Ahmed Abdel Tawab, Mohammad M. Alkherkhisy, Mohammed S. Abdulrahman

**Affiliations:** 1 Microbiology and Immunology Department, Faculty of Pharmacy (Boys), Al-Azhar University, Cairo, 11884 Egypt; 2 Botany and Microbiology Department, Faculty of Science, Al-Azhar University, Cairo, 11884 Egypt; 3 Department of Microbiology and Immunology, Faculty of Pharmacy, Ahram Canadian (ACU), Giza, Egypt; 4 Department of Microbiology and Immunology, Faculty of Pharmacy, Galala University, New Galala City, Suez Egypt; 5 Drug Microbiology Lab., Drug Radiation Research Department, National Center for Radiation Research and Technology (NCRRT), Egyptian Atomic Energy Authority (EAEA), Cairo, Egypt; 6 Department of Microbiology and Immunology, Faculty of Medicine, Al-Azhar University, Cairo, 11884 Egypt

**Keywords:** Biological synthesis, Starch/silver-selenium nanocomposite, Hemolytic activity, MIC, MBC, And Antibiofilm

## Abstract

**Background and objectives:**

Microbial cells capability to tolerate the effect of various antimicrobial classes represent a major worldwide health concern. The flexible and multi-components nanocomposites have enhanced physicochemical characters with several improved properties. Thus, different biological activities of biosynthesized starch/silver-selenium nanocomposite (St/Ag-Se NC) were assessed.

**Methodology:**

The St/Ag-Se NC was biosynthesized using *Cladosporium cladosporioides* CBS 174.62 (*C. cladosporioides*) strain. The shape and average particle size were investigated using scanning electron microscope (SEM) and high-resolution transmission electron microscope (HR-TEM), respectively. On the other hand, the St/Ag-Se NC effect on two cancer cell lines and red blood cells (RBCs) was evaluated and its hydrogen peroxide (H_2_O_2_) scavenging effect was assessed. Moreover, its effects on various microbial species in both planktonic and biofilm growth forms were examined.

**Results:**

The St/Ag-Se NC was successfully biosynthesized with oval and spherical shape and a mean particle diameter of 67.87 nm as confirmed by the HR-TEM analysis. St/Ag-Se NC showed promising anticancer activity toward human colorectal carcinoma (HCT-116) and human breast cancer (MCF-7) cell lines where IC_50_ were 21.37 and 19.98 µg/ml, respectively. Similarly, little effect on RBCs was observed with low nanocomposite concentration. As well, the highest nanocomposite H_2_O_2_ scavenging activity (42.84%) was recorded at a concentration of 2 mg/ml. Additionally, *Staphylococcus epidermidis* (*S. epidermidis*) ATCC 12,228 and *Candida albicans* (*C. albicans*) ATCC 10,231 were the highly affected bacterial and fungal strains with minimum inhibitory concentrations (MICs) of 18.75 and 50 µg/ml, respectively. Moreover, the noticeable effect of St/Ag-Se NC on microbial biofilm was concentration dependent. A high biofilm suppression percentage, 87.5% and 68.05%, were recorded with *S. epidermidis* and *Staphylococcus aureus* (*S. aureus*) when exposed to 1 mg/ml and 0.5 mg/ml, respectively.

**Conclusion:**

The biosynthesized St/Ag-Se NC showed excellent antioxidant activity, haemocompatibility, and anti-proliferative effect at low concentrations. Also, it exhibited promising antimicrobial and antibiofilm activities.

## Introduction

Antimicrobial resistance has emerged as a significant global health concern during the past twenty years [1]. Antimicrobial resistance is the capacity of a bacterium to withstand the impact of different antimicrobial agents. In this specific manifestation of resistance, bacteria possess the capacity to endure the impacts of medication that was formerly efficacious against them [2]. Multidrug resistance (MDR) is the occurrence of resistance to numerous medicines. Microorganisms possess a variety of resistance mechanisms, including innate resistance exhibited by particular microorganisms against certain antimicrobial agents, genetic changes, and acquired resistance acquired from other species [3]. The misuse of antibiotics has resulted in the emergence of numerous multidrug-resistant microorganisms [4, 5]. Another issue is cancer, which due to its high mortality rate, has risen to become a global health crisis of the twenty-first century. Every year, nearly 15 million people lose their lives because of lingering cancer cells [6]. Numerous chemotherapeutic medicines are already available for treating malignancies, but they all have significant negative effects on various human organs [7]. Thus, many of scientists have turned to use safe alternative to antimicrobial and anticancer drugs.

Nanotechnology have received much attention in the last period where have been used in medicine, pharmaceutical, chemistry and agriculture fields [8–15]. Nanoparticles (NPs) are defined as particles ranging from 1 to 100 nm in size [16, 17]. Their small size gives rise to a diverse range of atomic-level features [18–20]. Biopolymers are a big and promising group of polymers that have benefits like being biocompatible, biodegradable, and low in toxicity [21–23]. Starch is a consumable polysaccharide that finds application in several biological, nutritional, and food processing industries [24]. In addition, starch is employed as a reducing agent in the process of manufacturing copper NPs, which are then stabilized by chitosan polymers. Artificial implants utilize their antibacterial properties to serve as a covering that promotes wound healing [25]. Nanocomposites are novel materials that exhibit enhanced flexibility, as well as improved physical and chemical properties. These materials are characterized by their multifunctionality, as they are composed of several polymers [26]. Herein, this study aimed to evaluate the in vitro cytotoxicity of biosynthesized triple-component St/Ag-Se NC, identify its hemolytic and antioxidant activities, clarify its effect on microbial cell wall, and assess the destructive activity of these nanocomposite on both planktonic and biofilm forms of various bacterial as well as fungal strains.

## Materials and methods

### Biological synthesis of St/Ag-Se NC

#### Preparation of *Cladosporium* culture

One loop from *C. cladosporioides* culture, supplied from the Regional Center for Mycology and Biotechnology (RCMB), Al Azhar university, Cairo, Egypt, was grown in Sabouraud dextrose agar (SDA) and incubated 7 days. The *C. cladosporioides* was inoculated in 500 ml of double strength liquid malt extract medium with a pH of 5.6 in a 1000 ml Erlenmeyer flask and incubated stationary at 28 °C for 7 days [27].

### Synthesis of St/Ag-Se NC

After being cleaned with sterile water, the 10 gm mass cell of *C. cladosporioides* was reintroduced in 100 mL of purified water and incubated for one day at 28 °C and 120 rpm. The following procedure was utilised to create Ag-Se NPs after filtering the *C. cladosporioides* cell-free filtrate: For 24 h at 28 °C and 120 rpm, 2.0 mM silver nitrate and 2.0 mM sodium selenate were added to 100 mL of *C. cladosporioides* filtrate [28]. A deep brown color that developed in the cell-free filtrate implied Ag-Se NP production. Then, 400 mg of soluble starch was added, and the mixture was stirred for 60 min with a magnetic stirrer to create a mixture that covered silver-selenium nanocomposite (St/Ag-Se NC). The sample was dried in the oven at 120 °C for 24 h to obtain the product in a powder state in order to facilitate its use in further investigation.

### Characterization of St/Ag-Se NC

Using a UV-Vis. spectrophotometer (JASCO V-560) at certain wavelengths, the absorbance and optical properties of produced St/Ag-Se NC were examined. For Auto-zero reasons, a sample devoid of any metal ions was additionally included. All samples were initially screened for optical characteristics and to establish the fixed wavelengths used to calculate absorbance. As well, crystal structure and phase of the prepared St/Ag-Se NC were analyzed using X-ray diffraction analysis (XRD) analysis [29, 30]. Using the XRD-6000 lists, Shimadzu equipment, SSI, Japan, it was possible to assess the crystallization, crystallite size, and/or structure of the generated St/Ag-Se NC. The magnitude of the diffracted X-rays was measured using the diffracted angle 2θ [31]. To ascertain the mean size distribution of the produced NPs, measurements of dynamic light scattering (DLS) were made at the St. Barbara, California, USA facility using the DLS-PSS-NICOMP 380-ZLS particles sized system. One hundred microlitters of St/Ag-Se NC specimens were transferred to a temporary, tiny cuvette. Five procedures were carried out after equilibration at a temperature of 25 ± 2 °C for 2.0 min [32]. In addition, at the pH of synthesis, the surface charges of the biosynthesized St/Ag-Se NC were indirectly evaluated using a Malvern device Zeta potential analyzer, UK [31]. To clarify surface shape, border size, and the distribution of the generated St/Ag-Se NC surrounding starch, an analysis using SEM (ZEISS, EVO-MA10, Germany) was performed [33]. Finally, the shape, appearance, and average particle size of the produced St/Ag-Se NC were investigated using a HR-TEM (JEM2100, Jeol, Japan). St/Ag-Se NC sample used for TEM studies were drop-coated into carbon-coated TEM grids following dried in an incubator at 37.0 ± 2 °C [34].

### Evaluation of St/Ag-Se NC anti-proliferative effect

The 3-(4,5-dimethylthiazol-2-yl)-2,5-diphenyletetrazolium bromide (MTT) cytotoxicity assay was performed on HCT-116 and MCF-7 cancer cell lines using doxorubicin and imatinib as a control, respectively. This colorimetric assay evaluates cells proliferation based on the ability of live cells to enzymatically metabolize the MTT soluble salt to its insoluble purple formazan form that can be quantified spectrophotometrically at 570 nm [35]. Cells were detached from the T-75 flasks by addition of 1 ml trypsin/EDTA solution per flask and subsequently incubated for 3 min at 37 °C. Detached cells were then re-suspended in 10 ml complete growth medium, transferred to 15 ml Falcon tube, and centrifuged at 1000 rpm for 3 min to remove trypsin. The cell pellet was re-suspended in 10 ml growth medium with gentle agitation for well mixing. Using micropipette, 10 µl of cells suspension were placed on Hemocytometer to determine cell number using the inverted microscope. Using a handtally counter, the number of cells in this area of 16 squares was counted. The Hemocytometer was moved to another set of 16 corner squares and counting was carried on until all 4 corners squares were counted. The Hemocytometer is designed so that the number of cells in one set of 16 corner is equivalent to the number of cells X 10^4^. Therefore, once the total cell count has been obtained, the cell concentration (cell/ml) can be calculated as the following:

$$\mathbf{Total}\boldsymbol\;\mathbf{cell}\boldsymbol\;\mathbf{count}\boldsymbol\;\boldsymbol=\frac{\mathbf{Total}\boldsymbol\;\mathbf{cells}\boldsymbol\;\mathbf{counted}\boldsymbol\;\mathbf{in}\boldsymbol\;\mathbf4\boldsymbol\;\mathbf{squares}}{\mathbf{Number}\boldsymbol\;\mathbf{of}\boldsymbol\;\mathbf{squares}}\boldsymbol\times\mathbf{10}^{\mathbf4}\boldsymbol\;\left(\mathrm{cell}/\mathrm{ml}\right)$$

The cells suspension was diluted by complete medium to a concentration of 5 × 10^4^ cell/ml and 100 µl were pipetted into each well of 96 well plate (about 5000 cell/well) followed by incubation at 37 °C for 24 h to allow cells attachment. Cells were then treated with 100 µl of growth medium contains 0, 0.001, 0.01, 0.1, 1, 10, 100 and 1000 µg/ml of the tested nanocomposite in triplicate. After 72 h, media were removed from wells, 100 µl of MTT solution were added to each well, and incubated for 2 h at 37 °C. The excess MTT was then removed, 100 µl acidified isopropanol were added to each well, and the plates were incubated at 37 °C for 30 min with continuous shaking to dissolve the formed crystals. The developed purple color, that reflect the cells viability, was determined spectrophotometrically using Epoc-2 C micro-plate reader (BioTek, USA) at 570 nm. Results were expressed in terms of inhibition concentration fifty (IC_50_), the concentration required to inhibit cell growth by 50% relative to nanocomposite untreated cells, which calculated using Graph Pad Prism version 8.01, 2015 (GraphPad software, San Diego, USA). Through generating dose response curve by plotting the log concentration versus corresponding viability percentage [36].

### Assessment of St/Ag-Se NC hemolytic effect

The hemolytic activity of the biosynthesized nanocomposite was then examined as described by [37]: Briefly, 4 ml of fresh blood from healthy human were collected in EDTA tube, mixed with 8 ml of 0.2 M (Dulbecco’s) D-phosphate-buffered saline (D-PBS) solution (pH = 7), and centrifuged for 10 min at 10,000 rpm at 4 °C (for serum separation). For further rinsing, the RBCs in the pellet were resuspended in 8 ml phosphate buffer saline (PBS) followed by centrifugation at 10,000 rpm for 10 min. The obtained RBCs pellet was then suspended using 40 ml D-PBS. This suspension was divided into 3 parts, the 1st one or test sample subjected to the biosynthesized St/Ag-Se NC at different concentrations [0.25, 0.5, 1, and 2 mg/ml], the 2nd part was treated with triton X-100 (0.1% v/v) as a positive control, while the 3rd part was exposed to PBS as a negative control. All samples were incubated for 4 h at room temperature followed by vortex and centrifugation for 3 min at 10,000 rpm. In microtiter plate, for each 200 µl of the collected supernatant, the optical density (OD) was detected at 540 nm using an ELISA plate reader. The hemolysis percentages were then calculated as following:

$$\%\boldsymbol\;\mathbf H\mathbf e\mathbf m\mathbf o\mathbf l\mathbf y\mathbf s\mathbf i\mathbf s\boldsymbol\;\%=\frac{\mathbf M\mathbf e\mathbf a\mathbf n\boldsymbol\;\mathbf O\mathbf D\boldsymbol\;\mathbf o\mathbf f\boldsymbol\;\mathbf t\mathbf e\mathbf s\mathbf t\boldsymbol\;\mathbf s\mathbf a\mathbf m\mathbf p\mathbf l\mathbf e-\mathbf M\mathbf e\mathbf a\mathbf n\boldsymbol\;\mathbf O\mathbf D\boldsymbol\;\mathbf o\mathbf f\boldsymbol\;\mathbf n\mathbf e\mathbf g\mathbf a\mathbf t\mathbf i\mathbf v\mathbf e\boldsymbol\;\mathbf c\mathbf o\mathbf n\mathbf t\mathbf r\mathbf o\mathbf l}{\mathbf M\mathbf e\mathbf a\mathbf n\boldsymbol\;\mathbf O\mathbf D\boldsymbol\;\mathbf o\mathbf f\boldsymbol\;\mathbf p\mathbf o\mathbf s\mathbf i\mathbf t\mathbf i\mathbf v\mathbf e\boldsymbol\;\mathbf c\mathbf o\mathbf n\mathbf t\mathbf r\mathbf o\mathbf l-\mathbf M\mathbf e\mathbf a\mathbf n\boldsymbol\;\mathbf O\mathbf D\boldsymbol\;\mathbf o\mathbf f\boldsymbol\;\mathbf n\mathbf e\mathbf g\mathbf a\mathbf t\mathbf i\mathbf v\mathbf e\boldsymbol\;\mathbf c\mathbf o\mathbf n\mathbf t\mathbf r\mathbf o\mathbf l}\boldsymbol\times\mathbf{100}$$

### Detection of St/Ag-Se NC antioxidant activity

Hydrogen peroxide scavenging activity was performed to assess the St/Ag-Se NC antioxidant effect according to [38]. The experiment involved mixing 100 µl of the tested nanocomposite at concentrations of 0.5, 1, or 2 mg/ml with 0.3 ml of PBS (50 mM, pH = 7.4) and 0.6 ml of H_2_O_2_ solution (2 mM H_2_O_2_ in PBS, 50 mM, pH = 7.4). The solution was subjected to vortexing, and the absorbance at a wavelength of 230 nm was measured using a UV–Vis spectrophotometer (Systronics, AU-2701) after a duration of 10 min. Ascorbic acid served as the standard, whereas PBS (50 mM, pH = 7.4) was utilized as the blank. The H_2_O_2_ scavenging activity was quantified by calculating the percentage using the following formula:

$$\mathbf S\mathbf c\mathbf a\mathbf v\mathbf e\mathbf n\mathbf g\mathbf i\mathbf n\mathbf g\;\%=\frac{\mathrm{Ac}-\mathrm{As}}{\mathrm{Ac}}\times\mathbf{100}$$

Where Ac represents the absorbance of control (using PBS instead of the test sample) and As absorbance of St/Ag-Se NC or ascorbic acid.

### Effect of St/Ag-Se NC on microbial cell membrrane

Potassium (K^+^) ions leakage analysis was followed to clarify the St/Ag-Se NC effect on microbial culture. Fifty micolitters of 0.5 McFarland standard turbidity or 1 × 10^6^ spore/ml of bacterial or fungal spore (mold) suspension, respectively were inoculated onto 20 ml of nutrient broth and incubated for 24 h at 37 °C in case of bacteria or sabouraud dextrose broth (SDB) and incubated for 48 h at 28 °C in case of fungi. After centrifugation at 5000 rpm for 10 min, the supernatant discarded while the precipitated cells resuspended in 20 ml saline containing 100 µl of the biosynthesized nanocomposite at concentration 2 mg/ml, and incubated for 16 h at 37 °C. After centrifugation at 5000 rpm for 10 min, the supernatant was collected for determination of the released cytoplasmic K^+^ ion concentration using flame photometry. The experimental data were normalized against the data of untreated cell supernatant [control (C)] under the same conditions in absence of St/Ag-Se NC [39].

### Primary screening for St/Ag-Se NC antimicrobial activity

The agar well diffusion method was used for preliminary detection of St/Ag-Se NC, sodium selenite, silver nitrate, and starch antimicrobial effects [40]. Standardized concentrations, 0.5 McFarland of each overnight bacterial culture or 1 × 10^6^ spore/ml of each fungal spore suspension, were prepared and swabbed aseptically onto the Mueller-Hinton agar (MHA) plates for bacteria or SDA plates for fungi. Holes of 6 mm were then made on the agar using sterilized cork borer. Fifty microlitters of either starch, sodium selenite, silver nitrate, or St/Ag-Se NC were put in each hole under aseptic condition and kept at room temperature for 1 h. Chloramphenicol (30 µg/ml) and fluconazole (50 µg/ml) served as positive control for bacteria and fungi, respectively. The MHA plates were then incubated at 37 °C for 24 h while, the SDA plates were incubated at 28 °C for 48 h followed by inhibition zone diameters (IZDs) measurement [41]. In the current work, the antimicrobial activity of St/Ag-Se NC was assessed against standard strains of some Gram-positive bacteria including *Staphylococcus aureus* (*S. aureus*) ATCC 6538, *S. epidermidis* ATCC 12,228, and *Bacillus subtilus* (*B. subtilus*) ATCC 6633 and some Gram-negative bacteria including *Escherichia coli* (*E. coli*) ATCC 8739, *Pseudomonas aeruginosa* (*P. aeruginosa*) ATCC 9027, *Klebsiella pneumonia (K. pneumonia)* ATCC 13,882, *Proteus mirabilis* (*P. mirabilis*) ATCC 25,933, and *Salmonella typhi* (*S. typhi*) ATCC 14,028. On the other hand, the antifungal activity was assessed against some standard strains of molds including *Aspergillus niger* (A. *niger*) CBS 31.29, *Aspergillus. fumigatus (A. fumigatus)* CBS 106, and *Cladosporium herbarum* (Link) Fr. CBS 813.71 and some *Candida* species including *C. albicans* ATCC 10,231 and *Candida tropicalis* (*C. tropicalis*) ATCC 13,803.

### Quantitative antimicrobial tests

#### Determination of inhibitory concentrations

The St/Ag-Se NC minimum inhibitory concentration (MIC), minimum bactericidal concentration (MBC), and minimum fungicidal concentration (MFC) against selected bacterial and fungal standard strains were determined. Broth microdilution method according to Clinical Laboratory Standard Institute (CLSI) guidelines (2017) was applied for determination of MIC. Briefly, 50 µl of tryptic soya broth (TSB) for bacteria or SDB in case of fungi was added to each well of microtiter plate. In every raw, 50 µl from the prepared stock concentration of St/Ag-Se NC (2 mg/ml) in DMSO was added to the 1st well followed by two-fold serial dilution. Bacterial colonies from nutrient agar plates suspended into TSB and adjusted to 0.5 McFarland standard and fungal spore suspension at concentration 1 × 10^6^ was prepared. From this inoculum suspension, 10 µl was then added to every well followed by addition of the suitable broth to a final volume of 200 µl in each well. The TSB and SDB, containing DMSO without any tested compound, inoculated with the tested bacterial or fungal suspension, respectively were included as a positive control while the negative control containing TSB or SDB, DMSO, and St/Ag-Se NC without bacterial or fungal suspension was included. Following incubation 37 °C for 24 h for bacteria or at 28 °C for 48 h for filamentous fungi, 50 µl of MTT (0.2 mg/ml) was added followed by incubation at 37 °C for 30 min. The lowest concentration of the tested compound, in the well showed no color change from yellow to pink, was scored as the MIC [42, 43]. The suspension from the MIC containing well and other wells containing the higher concentrations of the tested compound were subcultured on MHA or SDA plates for determination of MBC or MFC, respectively. On completion of incubation, the lowest concentration of the tested compound causing no growth was considered as the MBC for bacteria and MFC for fungi. Subsequently, each microbial strain tolerance level was calculated according to the equation described by [44]:

$$\mathrm{Tolerance}\;\mathrm{level}\;=\frac{\mathrm{MBC}}{\mathrm{MIC}}$$

The tolerance values of the tested nanocomposite ≤ 2 indicated their bactericidal mode of action with consequent complete microbial cells eradication abilities. Conversely, tolerance values ≥ 4 referred to their bacteriostatic activity with subsequent ability for only bacterial growth inhibition [45].

#### Biofilm destructive assay

The crystal violet method in microtiter plates was used to evaluate the effect of the biosynthesized St/Ag-Se NC at (1, 0.5, 0.25, 0.125, and 0.0625 mg/ml), against the biofilm forming clinical isolates of *S. aureus, B. subtili, S. epidermidis, E. coli, P. mirabilis, P. aeruginosa, K. pneumonia, S. typhi, A. niger, A. fumigatus, C. albicans*, and *C. herbarum* in 96 well microtitre plate. Each well was inoculated with 180 µl of Luria-Bertani (LB) broth and 10 µl of either bacterial suspension of 0.5 McFarland or 1 × 10^6^ spore/ml of each fungal spore suspension. Ten milliliters of the tested St/Ag-Se NC concentration suspended in DMSO was then added to each well. The plates were incubated at 37 °C for 16 h in case of bacteria or 35 °C for 24 h for fungi and cultures without any tested compound were taken as control. The broth containing suspended cells were gently discarded, wells washed 4 times with 10 µl PBS (pH = 7). To each well, 200 µl of 0.4% crystal violet was added followed by incubation for 20 min at 37 °C. The wells washed 3 times with PBS to rinse off the excess crystal violet while, the stain retained by the biofilm was solubilized in 200 µl of 33% acetic acid and incubated for 30 min. The OD, caused by bacterial or fungal biofilm, was measured at 590 nm using an ELISA microtitre plate reader (Multiskan Ascent, Labsystems, Helsinki, Finland). The percentage of biofilm change was calculated according to Hwang et al. [46] using the equation:

$$\mathbf B\mathbf{io}\mathbf{fi}\mathbf l\mathbf m\boldsymbol\;\mathbf c\mathbf h\mathbf a\mathbf n\mathbf g\mathbf e\boldsymbol\;\left(\boldsymbol\%\right)=1-\frac{\mathbf O\mathbf D\boldsymbol\;590\;\mathbf o\mathbf f\boldsymbol\;\mathbf c\mathbf e\mathbf{lls}\boldsymbol\;\mathbf t\mathbf r\mathbf e\mathbf a\mathbf t\mathbf e\mathbf d\boldsymbol\;\mathbf w\mathbf i\mathbf t\mathbf h\boldsymbol\;\mathbf n\mathbf a\mathbf n\mathbf o\mathbf c\mathbf o\mathbf m\mathbf p\mathbf o\mathbf s\mathbf i\mathbf t\mathbf e}{\mathbf O\mathbf D\boldsymbol\;590\;\mathbf o\mathbf f\boldsymbol\;\mathbf u\mathbf n\mathbf t\mathbf r\mathbf e\mathbf a\mathbf t\mathbf e\mathbf d\boldsymbol\;\mathbf c\mathbf e\mathbf l\mathbf l}\boldsymbol\times100$$

### Statistical analysis

In the current work, the IC_50_ values were calculated using Graph Pad Prism version 8.01, 2015 (GraphPad software, San Diego, USA) through generating dose response curve by plotting the log concentration versus corresponding viability percentage. In addition, calculation of percentages, mean, standard deviation (SD), and *P-*value values was conducted using Microsoft Excel® ver. 2016.

## Results

### Characterization of St/Ag-Se NC

#### XRD analysis and UV-Vis. Spectroscopy

**Fig. 1:**
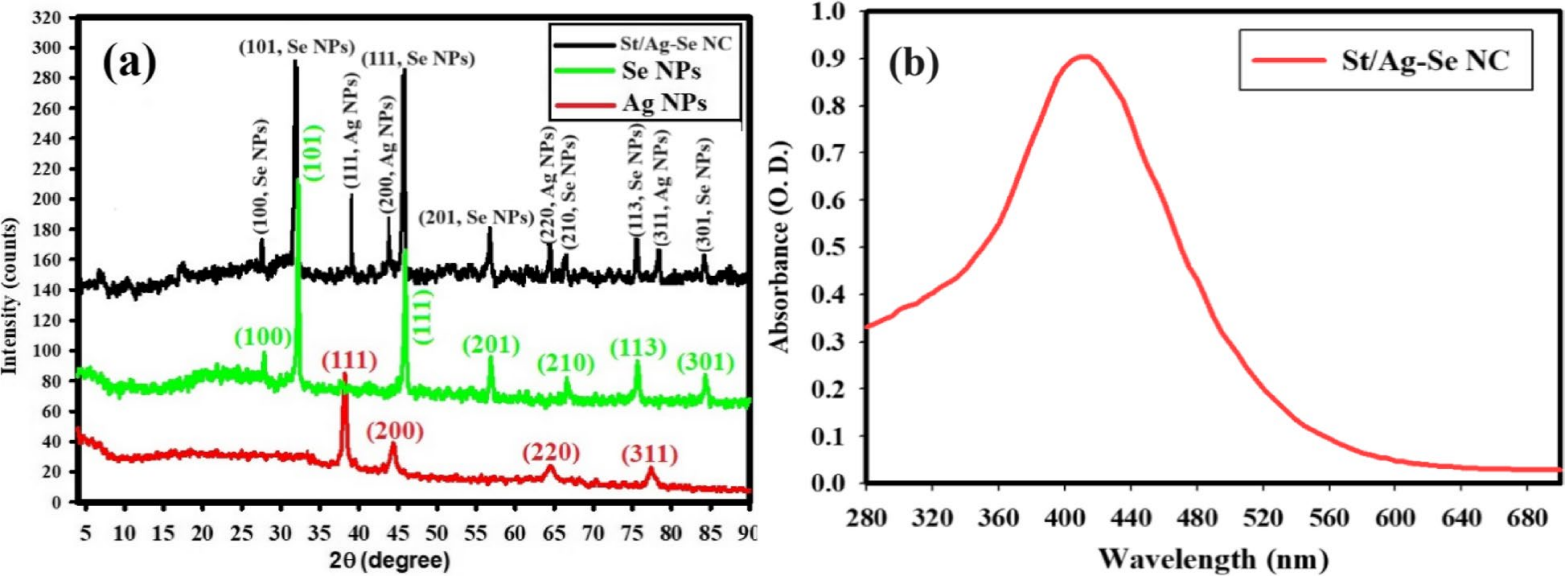
Crystallinity and optical property of the biosynthesized St/Ag-Se NC, where (**a**) XRD analysis and (**b**) UV-Vis. spectroscopy

Figure 1a displays the XRD pattern of the biosynthesized St/Ag-Se NC, together with the patterns of Ag NPs and Se NPs for comparison. The pattern clearly indicates the absence of distinct peaks for the first precursors, namely silver nitrate or sodium selenite. The XRD analysis verified the presence of a nano-complex composed of silver-selenium (Ag-Se) and starch (St). Figure 1a displays XRD peaks of Ag NPs, specifically at 2θ angles of 38.88^o^, 43.09^o^, 65.90^o^, and 77.31^o^. These peaks correspond to the (111), (200), (220), and (311) Bragg’s reflections, respectively, and are in agreement with the JCPDS-ICDD 04-0783 card from the Joint Committee on Powder Diffraction Standards (JCPDS). Figure 1a presents the XRD diffraction peaks of Se NPs, indicating the diffraction features at specific angles (2 θ) of 27.78^o^, 33.10^o^, 46.67^o^, 57.98^o^, 67.10^o^, 75.66^o^, and 84.94^o^. These angles correspond to the Bragg’s reflections at (100), (101), (111), (201), (210), (113), and (301) crystal planes, respectively. The peaks exhibited a resemblance to the JCPDS of Se NPs, specifically matching the standard card JCPDS File No 06-0362. Furthermore, the XRD results for all the generated St/Ag-Se NCs exhibit diffraction features at 2θ that are similar to those observed for both Ag NPs and Se NPs. This suggests that the produced St/Ag-Se NCs possessed a crystalline structure.

The deep brown color observed in the generated substance can be attributed to the activation of the surface Plasmon resonance of biogenic St/Ag-Se NC, providing a reliable spectroscopic signal of their existence. The experimental peak in the spectra (Fig. 1b) was clearly observed as a result of the O.D. (0.906; diluted three times). The UV-Vis analysis revealed that the produced St/Ag-Se NC were minuscule and detectable at a wavelength of 410.0 nm.

#### DLS analysis and Zeta potential

**Fig. 2:**
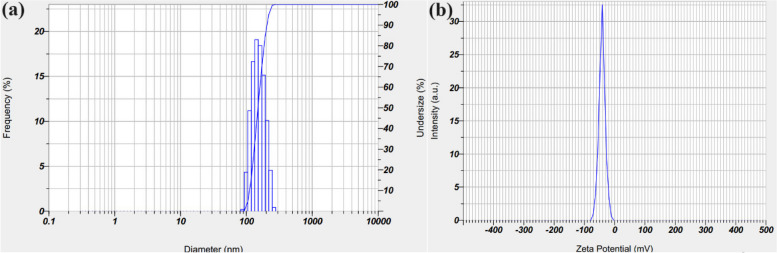
Particle size distribution and surface charge determination of the biosynthesized St/Ag-Se NC, where (**a**) DLS analysis, and (**b**) Zeta potential

 DLS analysis was performed to determine the particle size distribution in order to evaluate the distribution of particle sizes and calculate the mean size of particles for St/Ag-Se NC. Figure 2a illustrates the results, which are 190.89 nm. The Zeta potential of the produced St/Ag-Se NC was examined at the pH of the synthesis (7.2), as depicted in Fig. 2b. According to the current work, the biosynthesized St/Ag-Se NC maintains a negative surface Zeta potential at the studied pH of the synthesis. Furthermore, due to the negative charge of starch, the Zeta potential of the preparation at a neutral pH of 7.2 was measured to be -41.9 mV, as shown in Fig. 2b.

#### SEM analysis

**Fig. 3:**
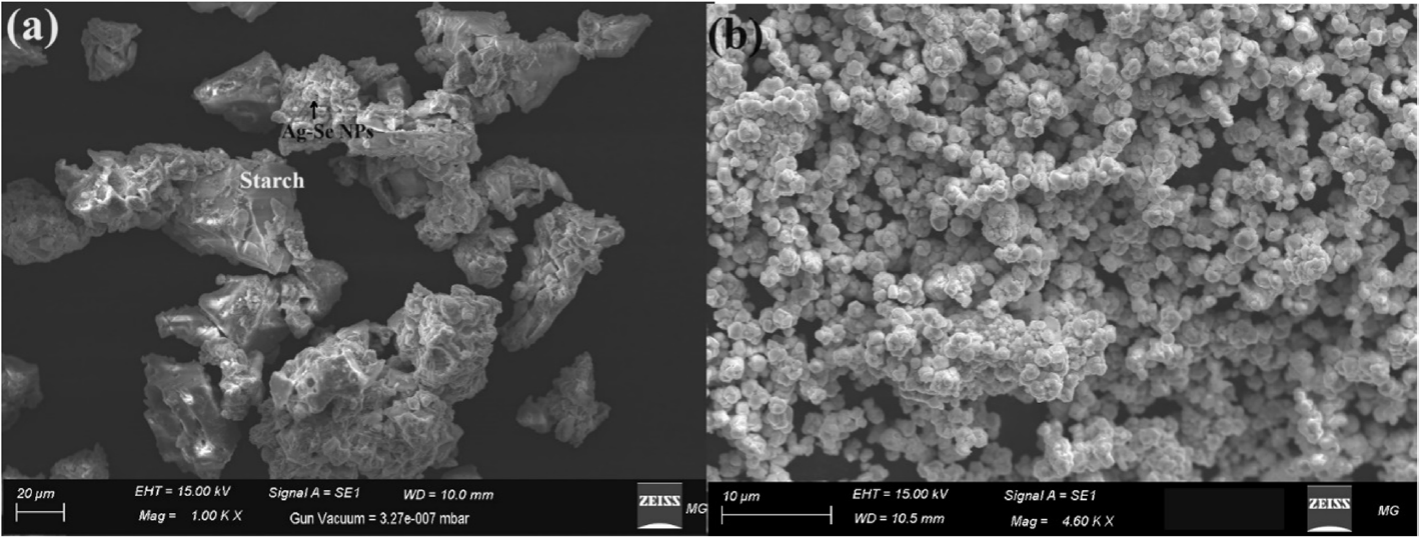
Morphological surface determination, where (**a**) SEM imaging of the prepared St/Ag-Se NC, and (**b**) SEM imaging of the biosynthesized St/Ag-Se NC at a high magnification

Fig. 3 depicts the appearance, shape, and morphological characteristics of the biosynthesized St/Ag-Se NC. Figure 3a shows that bimetallic Ag-Se NPs, which appeared as brilliant particles, were evenly spread throughout starch polymer. According to Fig. (3b), Ag and Se NPs were dispersed uniformly throughout the starch as a clear spherical NPs crowded across the starch polymer.

#### HR-TEM analysis

**Fig. 4:**
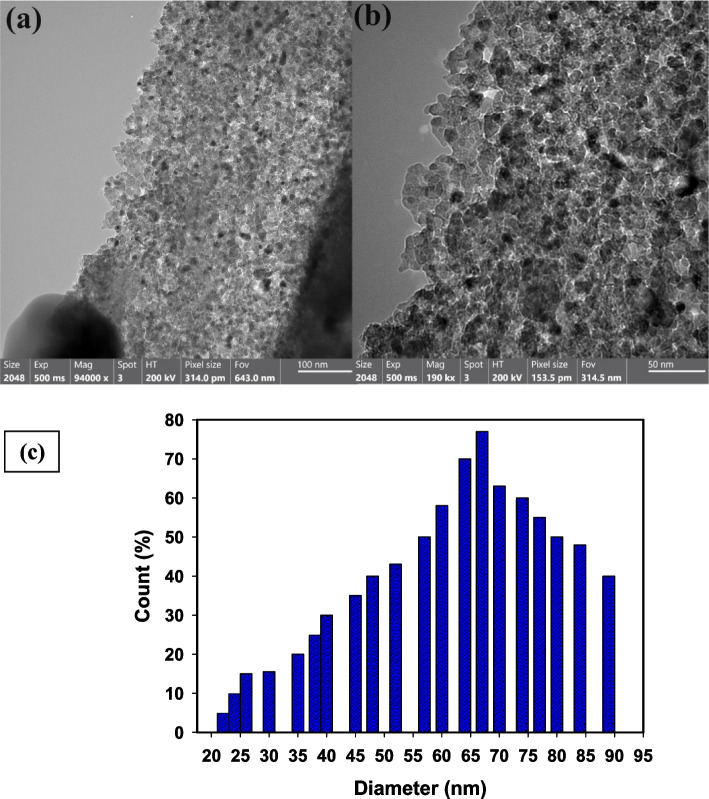
Mean particle size, and shape, of the biosynthesized St/Ag-Se NC, where (**a**) HR-TEM imaging of the biosynthesized St/Ag-Se NC, (**b**) at a high magnification and (**c**) the histogram of the particle size distribution of the biosynthesized St/Ag-Se NC

 HR-TEM analysis was used for characterization of shape and size of the biosynthesized St/Ag-Se NC (Fig. 4). It was possible to determine the mean size of the particles and see how the biosynthesized St/Ag-Se NC appeared. Additionally, data from HR-TEM and DLS measurements were compared. The produced St/Ag-Se NC have a variety of forms, including oval and spherical morphologies, as seen in HR-TEM Fig. 4a. The biosynthesized St/Ag-Se NC varied in size from 22.6 to 89.45 nm, with a mean diameter of 67.87 nm, as shown in Fig. 4b. The solid lines in Fig. 4c represent the size distribution histogram of the biosynthesized St/Ag-Se NC and confirms the size distribution of the biosynthesized St/Ag-Se NC.

### Anti-proliferative activity of Ag St/Ag-Se NC

**Fig. 5:**
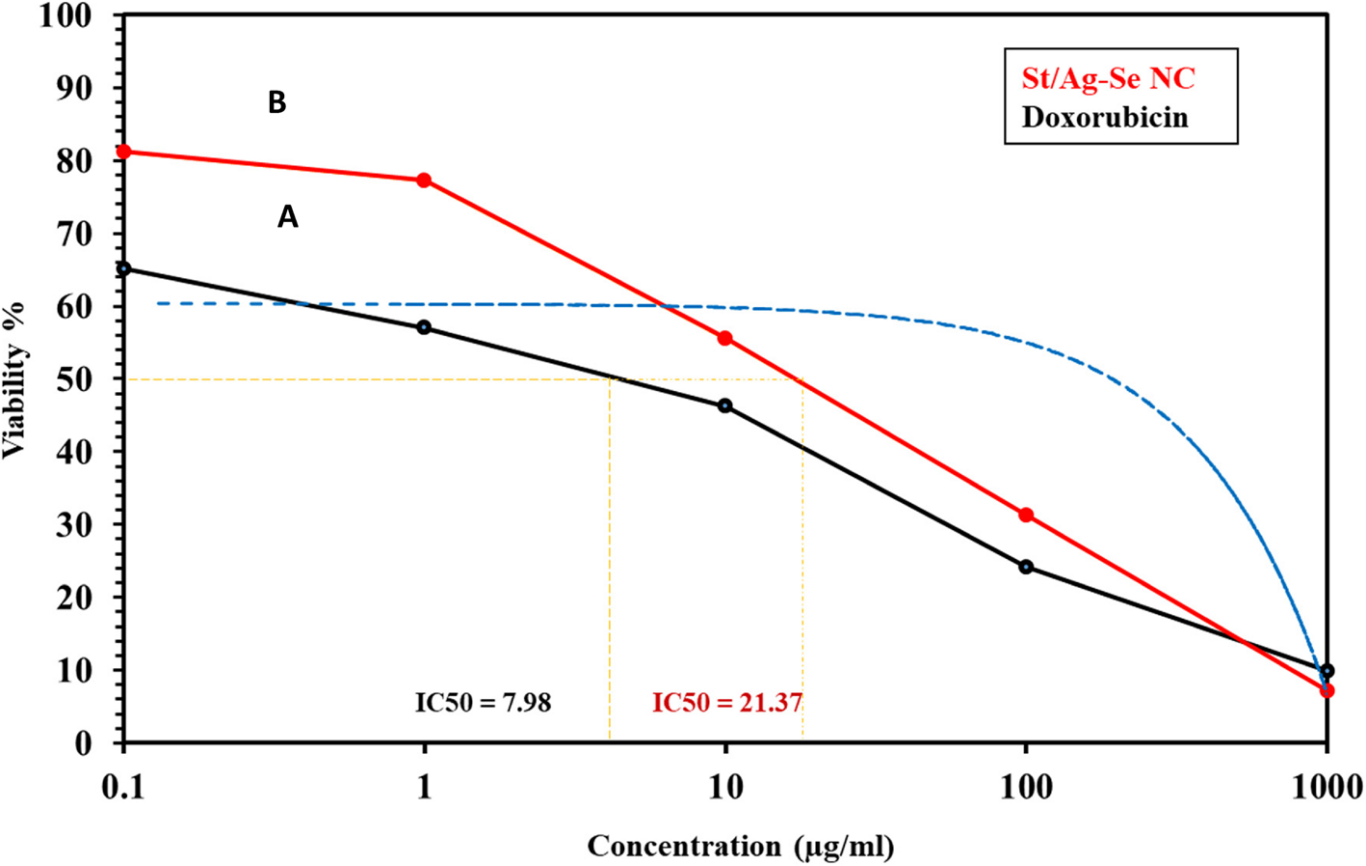
Anti-proliferative activity of doxorubicin (**A**) and St/Ag-Se NC (**B**) against HCT-116 cell line

**Fig. 6:**
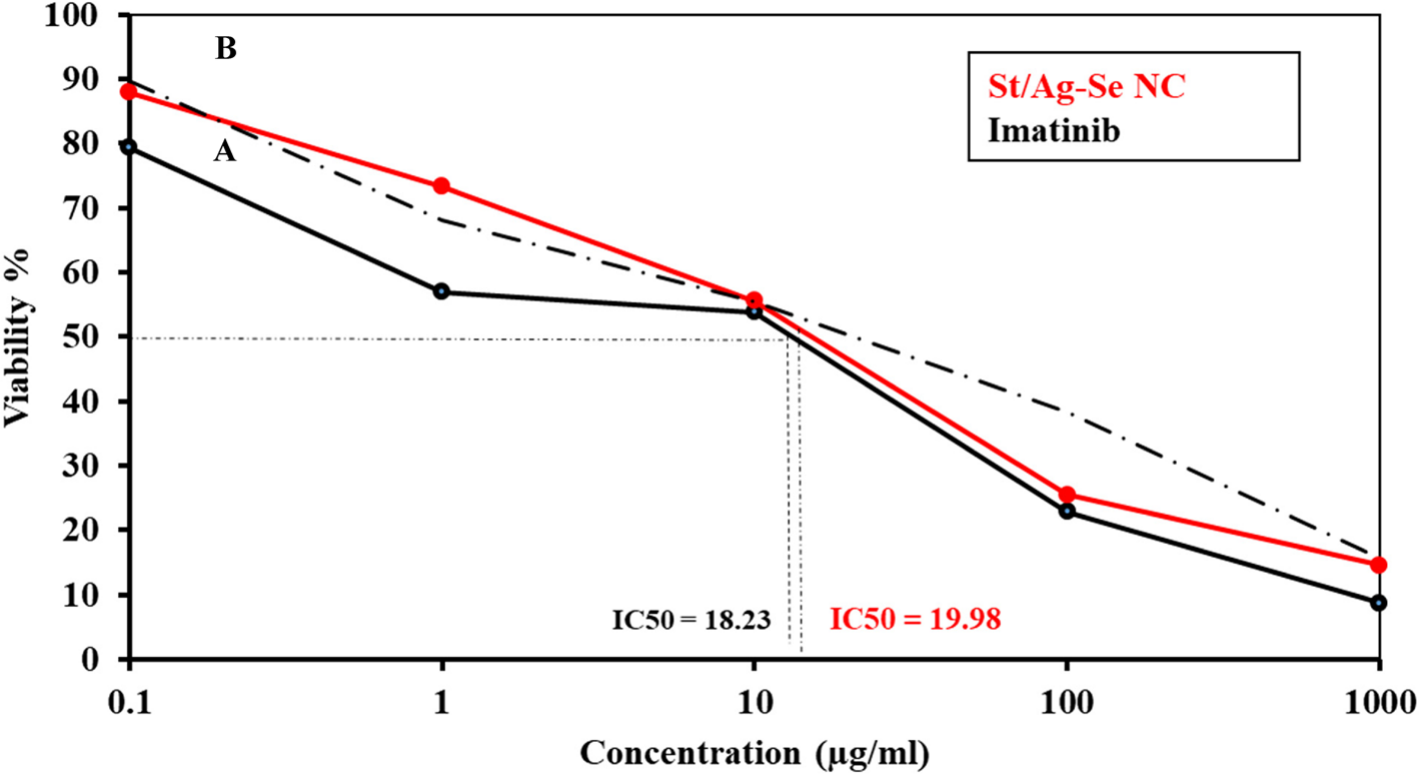
Anti-proliferative activity of imatinib (**A**) and St/Ag-Se NC (**B**) against MCF-7 cell line

 Exposure of the malignant colon cell line HCT-116 and breast cancer cell line MCF-7 to different concentrations of St/Ag-Se NC, at a range of 0.1 µg/ml–1 mg/ml, illustrated their anti-proliferative outcome. The viability percentages of both cell lines were decreased with the increased concentrations of St/Ag-Se NC, reaching the least value at 1 mg/ml, in comparison with doxorubicin and imatinib that were used as a controls in case of HCT-116 and MCF-7 cell lines, respectively. Moreover, the IC_50_ was markedly higher with St/Ag-Se NC than doxorubicin against HCT-116 cell line, with values 21.37 and 7.98 µg/ml, respectively (Fig. 5). While, the IC_50_ was slightly higher with St/Ag-Se NC than imatinib against MCF-7 cell line, with values of 19.98 and 18.23 µg/ml, respectively (Fig. 6).

### Blood toxicity of St/Ag-Se NC

**Fig. 7:**
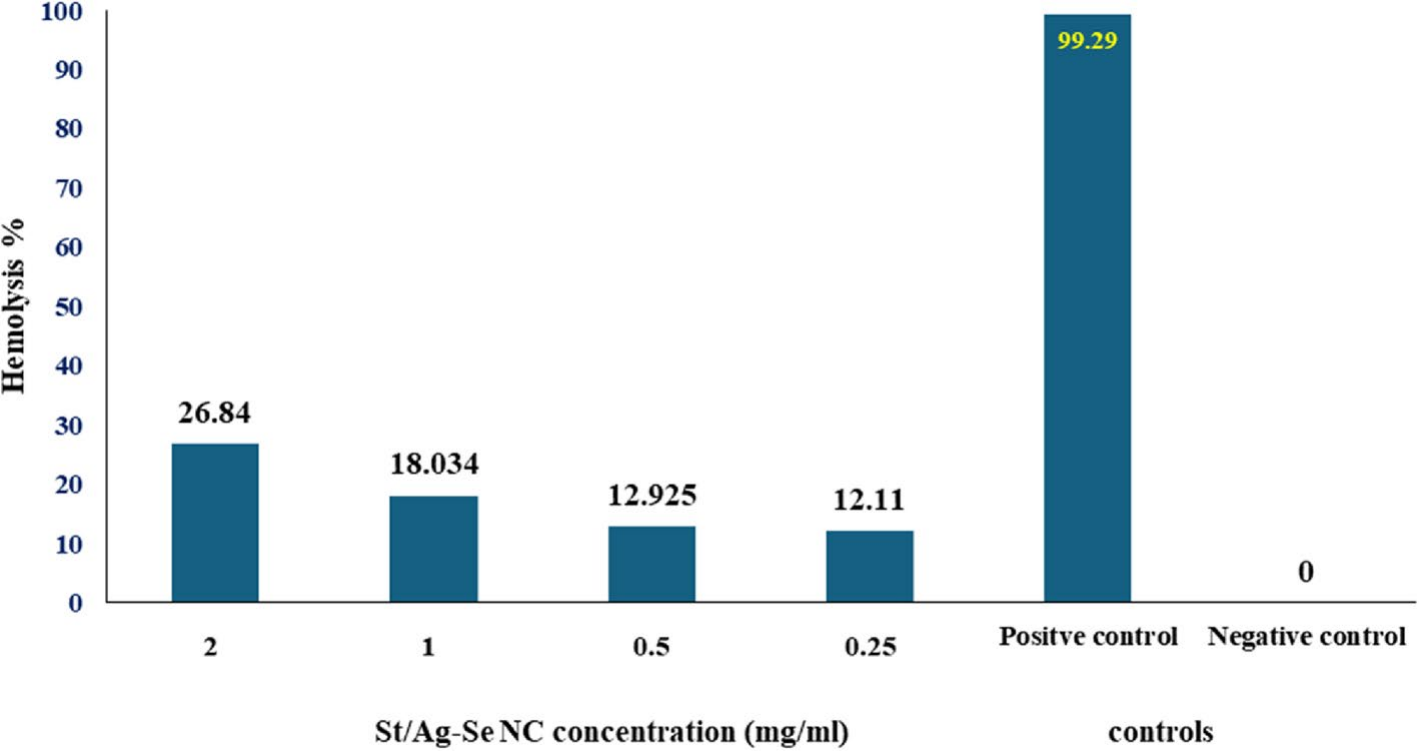
Hemolytic activity of St/Ag-Se NC at different concentrations in comparison to the positive control (triton X-100) and negative control (PBS)

 Study of St/Ag-Se NC hemolytic activity revealed decreased degree of hemolysis as the nanocomposite concentration decrease. A low hemolysis percentage was recorded at nanocomposite concentration of 0.25 mg/ml in comparison to triton X-100 and PBS (Fig. 7).

### Antioxidant activity of St/Ag-Se NC

**Fig. 8:**
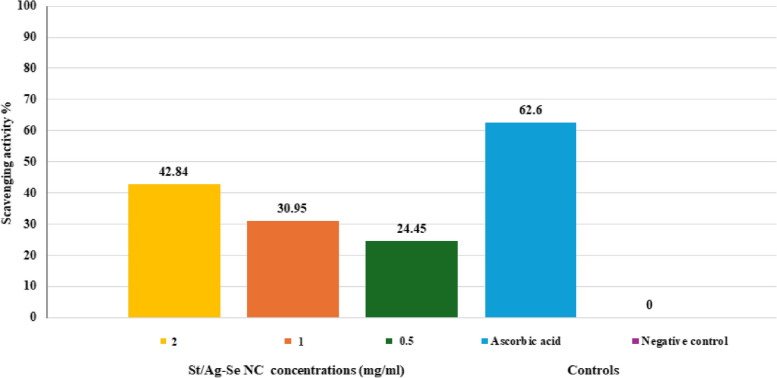
H_2_O_2_ scavenging activity of biosynthesized St/Ag-Se NC at different concentrations in comparison to positive control (ascorbic acid) and negative control (PBS)

 The results confirmed the increased H_2_O_2_ scavenging activity of the biosynthesized St/Ag-Se NC with the increased concentration, as compared with ascorbic acid (50 µg/ml) that served as positive control. The highest scavenging activity (42.84%) was observed at a concentration of 2 mg/ml St/Ag-Se NC in comparison to 62.5% recorded by ascorbic acid (Fig. 8).

### Biosynthesized St/Ag-Se NC potassium leakage effect

**Fig. 9:**
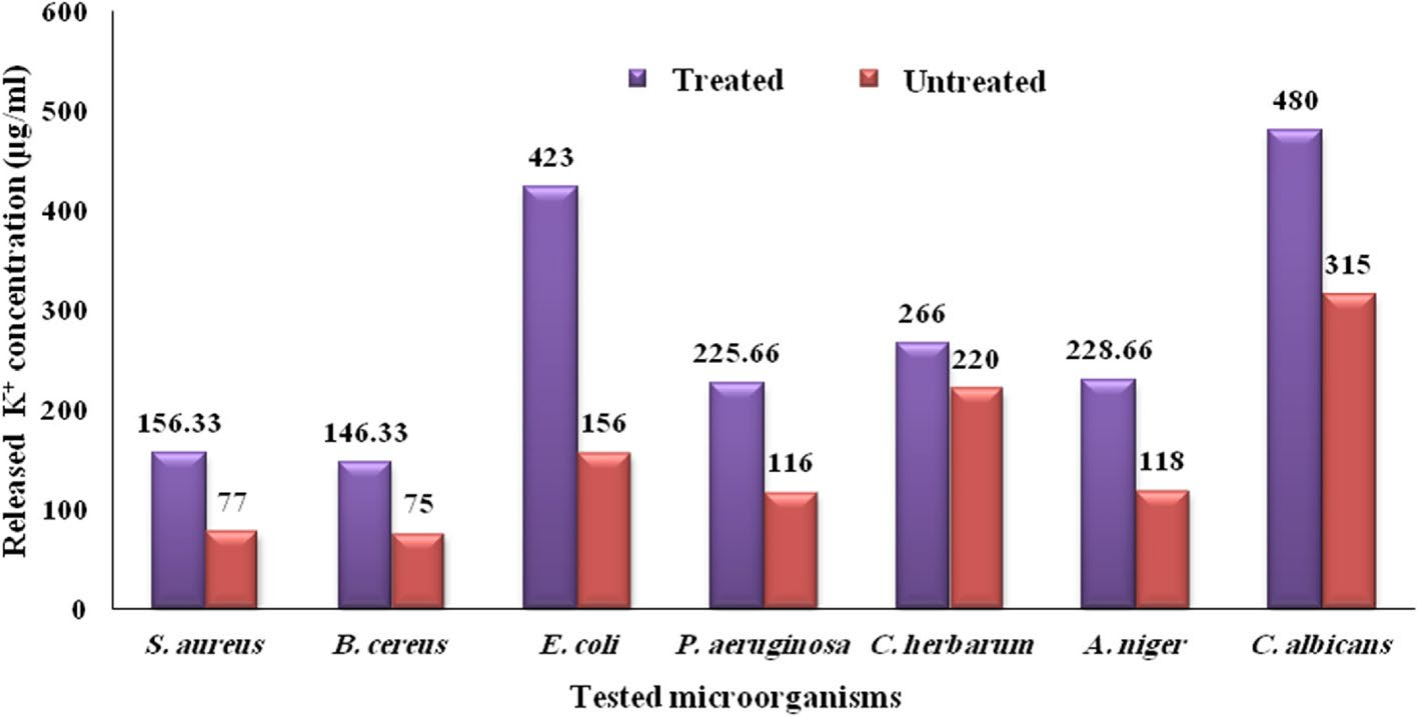
Amount of K **+** released from St/Ag-Se NC treated and untreated microbial cells

The potassium leakage assay was clarify the effect of biosynthesized St/Ag-Se NC, at concentration of 2 mg/ml, on microbial cell membrane. The highest concentration of potassium was detected with *E. coli* and *C. albicans*. This observation may prove, with other results showed in (Fig. 9), the highest impact of St/Ag-Se NC on both bacterial and fungal cells membrane. Statistical analysis of our observations showed significant difference between treated and untreated samples with *P* value 0.021.

### Antimicrobial activity of St/Ag-Se NC

#### Agar well diffusion assay

**Fig. 10:**
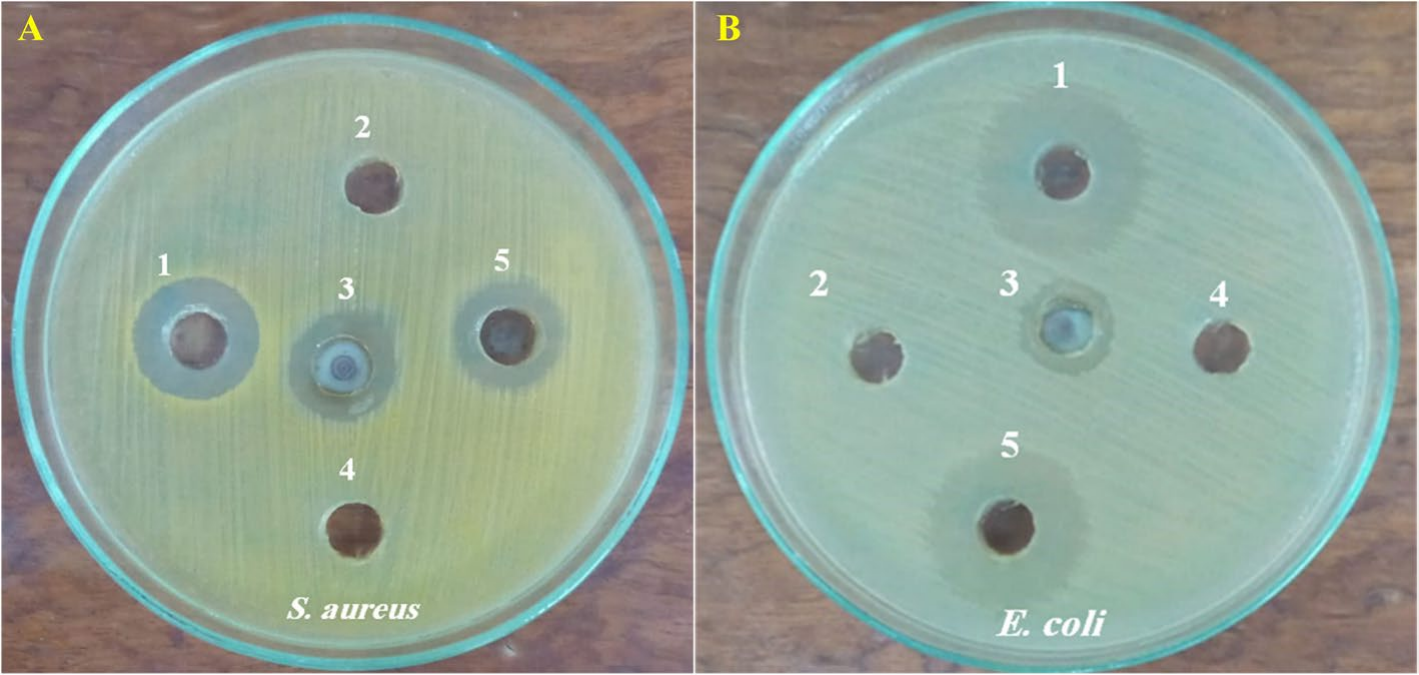
Antimicrobial activity of St/Ag-Se NC using agar well diffusion method against S. aureus ATCC 6538 (**A**) and E. coli ATCC 8739 (**B**). Wells 1; Tested St/Ag-Se Nc, 2; Sodium selenite, 3; AgNO_3_, 4; Starch, and 5; Positive control, chloramphenicol

**Fig. 11:**
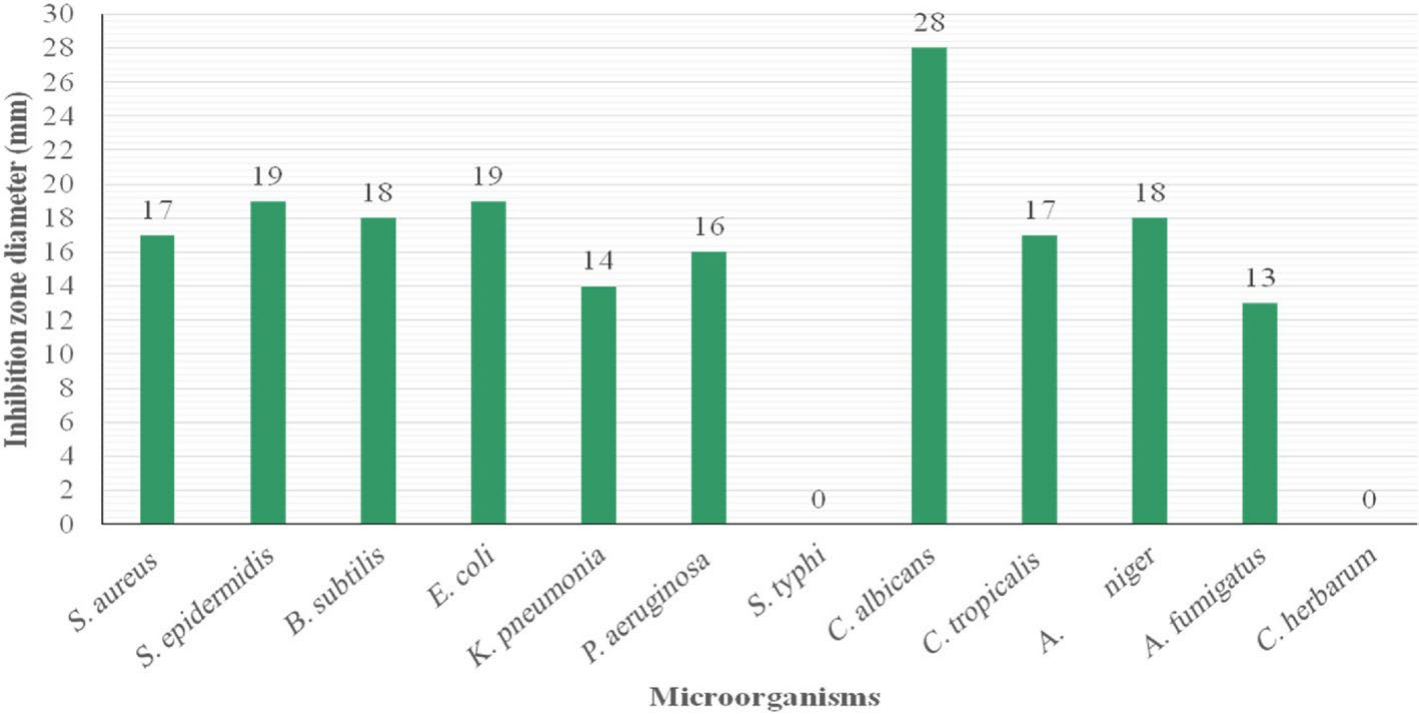
Various IZDs caused by St/Ag-Se NC, in agar well diffusion assay, against the tested microbial strains

In the present study, St/Ag-Se NC at a concentration of 2 mg/ml showed antimicrobial activity against most of the tested bacterial and fungal standard strains including *S. aureus*, *S. epidermidis*, *B. subtilus*, *E. coli*, *P. aeruginosa*, *K. pneumonia*, *P. mirabilis*, A. *niger*, *A. fumigatus*, *C. albicans*, and *C. tropicalis*. Unfortunately, *S. typhi* and *C. herbarum* not affected by the nanocomposite at the applied concentration. Moreover, the highest antibacterial activity was observed against *S. aureus, E. coli*, and *S. epidermidis* with IZDs of 21, 21, and 19 mm, respectively in comparison to chloramphenicol (30 µg/ml) that displayed IZDs 28, 24, and 24 mm, respectively. On the other hand, the maximum antifungal activity was observed against *C. albicans* and showed IZD 28 mm, in comparison to fluconazole (50 µg/ml) with IZD 32 mm (Figs. 10 and 11).

#### Inhibitory concentrations of St/Ag-Se NC

**Fig. 12:**
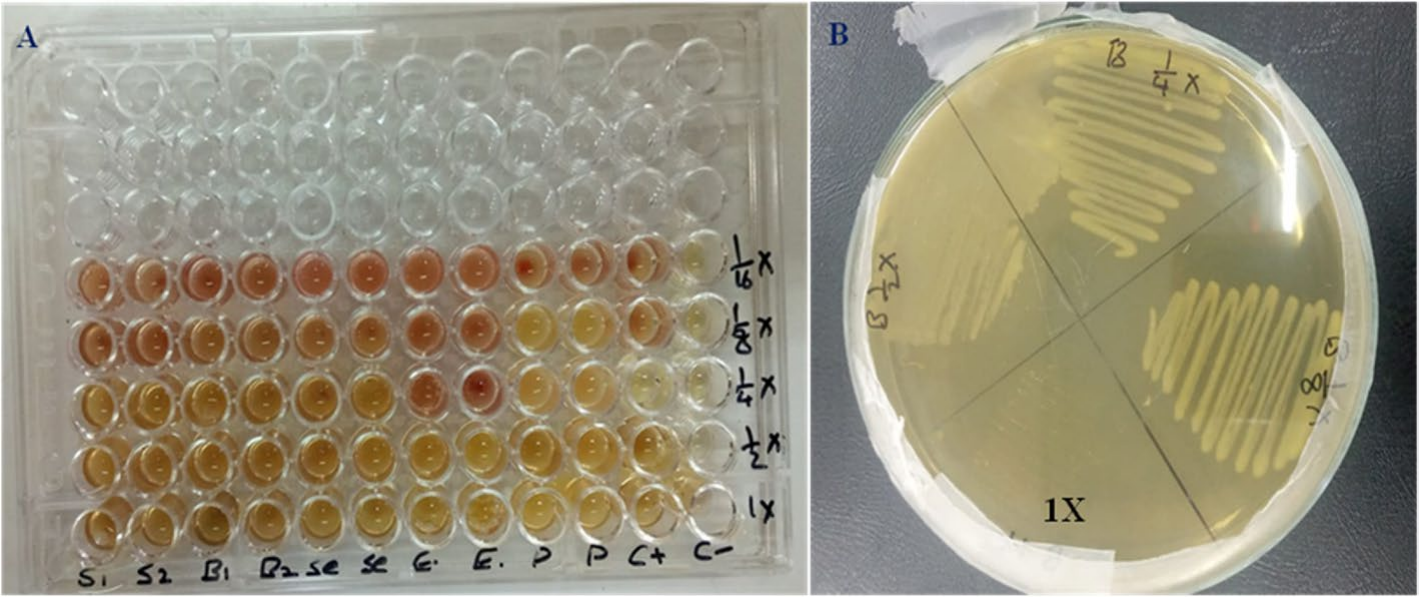
Representative St/Ag-Se NC antimicrobial activity. **A** Broth microdilution assay. Wells S; S. aureus ATCC 6538, B; B. subtilis ATCC 6633, Se; S. epidermidis ATCC 12,228, E; E. coli ATCC 8739, P. aeruginosa ATCC 9027, C+; Positive control; C-; Negative control, and 1X-1/16X; Serial dilutions. **B **Minimum bactericidal concentration assay. 1X dilution showed no growth and recorded as MBC

The highest antibacterial activity of St/Ag-Se NC was observed against *S. epidermidis* with lowest MIC (18.75 µg/ml) followed by *S. aureus, E. coli*, and *B. subtilis* with MIC of 25 µg/ml for each. On the other hand, the maximum antifungal activity was observed against *C. albicans* where the MIC was 50 µg/ml. In addition, the MBC was observed with nanocomposite concentration of 25 µg/ml against *S. aureus, B. subtilis*, and *E. coli*. Similarly, the MFC was noticed as 100 µg/ml against all of the tested fungi while, the microbial tolerance was unity with *S. aureus, B. subtilis, E. coli, C. tropicalis*, and *A. fumigatus* (Fig. 12 and Table 1).

**Table 1:** The MICs, MBCs, and MFCs of St/Ag-Se NC and their tolerance by various microbial isolates

Tested Microorganism	Antimicrobial activity			Tolerance
	MIC^a^	MBC	MFC	
	µg/ml			
***S. aureus*ATCC 6538**	**25**	**25**	-	1
***S. epidermidis*ATCC 12,228**	**18.75**	**50**	-	2.67
***B. subtilis*ATCC 6633**	**25**	**25**	-	1
***E. coli*ATCC 8739**	**25**	**25**	-	1
***K. pneumonia*ATCC 13,882**	**75**	**200**	-	2.67
***P. aeruginosa*ATCC 9027**	**75**	**100**	-	1.33
***P. mirabilis*ATCC25933**	**100**	**200**	-	2
***C. albicans*ATCC 10,231**	**50**	-	**100**	2
***C. tropicalis*ATCC 13,803**	**100**	-	**100**	1
***A. niger*CBS 31.29**	**75**	-	**100**	1.33
***A. fumigatus*CBS 106**	**100**	-	**100**	1

^a^*MIC* Minimum inhibitory concentration, *MBC* Minimum bactericidal concentration, and *MFC* Minimum fungicidal concentration

### Anti-biofilm activity of St/Ag-Se NC

 The effect of St/Ag-Se NC, at different concentrations, on various bacterial and fungal biofilm was noticeable. High biofilm changes, at concentration 1 mg/ml, were observed with *S. epidermidis* and *B. subtilis* at 87.5% and 82.5%, respectively. In addition, *S. aureus, E. coli*, and *P. mirabilis* showed high changes in their biofilms, 68.05%, 66.12%, and 65.37%, respectively, when treated with 0.5 mg/ml of St/Ag-Se NC (Table 2).

**Table 2:** Microbial biofilm suppression (%) by different concentrations of St/Ag-Se NC

Tested microorganisms	Biofilm change (%)/St/Ag-Se NC concentration (mg/ml)				
	1	0.5	0.25	0.125	0.0625
***S. aureus***	77.37	68.05	49.94	19.59	16.38
***B. subtilis***	82.68	55.37	38.43	16.07	4.945
***S. epidermidis***	87.55	73.44	36.34	16.94	12.14
***E. coli***	77.78	66.12	54.16	26.24	7.737
***P. mirabilis***	73.08	65.37	52.17	18.27	4.662
***P. aeruginosa***	70.48	59.55	48.83	15.85	10.35
***K. pneumonia***	76.19	57.16	47.35	18.78	6.502
***S. typhi***	27.53	19.84	18.07	14.51	6.307
***A. niger***	31.98	27.97	24.14	21.28	12.05
***A. fumigatus***	27.45	22.12	13.16	11.96	4.57
***C. albicans***	77.76	63.35	32.67	25.13	13.97
***C. herbarum***	76.04	61.15	59.44	30.71	7.07

## Discussion

Nanocomposite material contains number of phases with at least one dimension of which in nanometer scale. These dimension enhance materials characters as it creates a very vital phase interfaces [47, 48]. The usual availability, best antimicrobial activities, and low human cytotoxicity of Ag NPs reflect their extensive applications in biomedical field [49, 50]. Additionally, selenium is one of the micronutrients essential for human’s healthy body with anti-inflammatory and redox activities. It is essential for activation of human’s immunity and nervous system. As well, cardiomyopathies and other several diseases has been alleviated with proper selenium supplements [51].

The intensity of the deep brown color corresponded to the capacity to biosynthesize St/Ag-Se NC. The physical characteristics, such as strength, size, surface structure, and dielectric properties, of NPs play a crucial role in surface plasmon resonance (SPR). It is worth mentioning that a little 2θ shift was observed, which may be attributed to the formation of St/Ag-Se NC. The lack of peaks at 2θ = 31.30^o^, 32.62^o^, and 33.68^o^ suggests that the synthesized Ag NPs, Se NPs, and Ag-Se NPs were pure and did not contain any AgO NPs or SeO NPs [52–54]. Furthermore, the XRD results demonstrate that the synthesized possess a high degree of crystallinity, which enhances their suitability for various applications [41].

The produced St/Ag-Se NC, in the present study, were uniformly distributed with wide size and the same spherical shape, according to a comparison of the literature about the morphological shape and analysis of elements. Bimetallic silver and gold core-shell NPs were created by [55] using the citrate reduction process at various pH levels and temperatures. The acquired morphological form and boundary size suggested that they had a size varied from 50 to 65 nm and seem as spherical particles, which means that both pH and temperature play a crucial role in the creation process. Finally, our findings were compared with the newly published studies [56–59].

As well, the produced St/Ag-Se NC varied in size and were mostly spherical in form, according to a comparison of average particle size and shape in the literature. Castro-Longoria et al. [60] mycosynthesized silver, gold, and silver-gold bimetallic NPs, where the shape of the NPs was found to be mainly spherical with a mean diameter of 11.0 nm for silver and 32.0 nm for gold.

Although different morphologies may be noticed owing to the synthetic process from extract, the anisotropic shape had been recorded, the created forms in that work [60], may be varied as the shape of extracted NPs was roughly circular or ellipsoidal in all cases. Due to the use of only one reducing and capping agent, a constant form is seen in our investigation. Finally, our findings were compared with the newly published studies [61–64].

It is important to note that the starch used, which served as a suitable capping and stabilizing agent, was responsible for the creation of the mono-distributed NPs [65]. Dynamic light scattering analysis determines the hydrodynamic diameter of NPs surrounded by water molecules, which leads to larger sizes of the capped NPs. On the other hand, HR-TEM analysis calculates the actual particle size of the substance without the solvent layer. Consequently, it is typical for DLS size measurements to exhibit higher values compared to HR-TEM measurements [66]. The NPs that were produced were highly dispersed in a restricted range of sizes due to the scientific validity of DLS, which significantly improved their characteristics and applications [67–70].

Upon comparing our combination St/Ag-Se NC with existing literature on particles of intermediate size and form, we discovered that our particles were poly-dispersed, exhibited size variation, and predominantly had spheroidal shapes. Diverse forms may have been developed in that work. While the majority of the newly biosynthesized St/Ag-Se NCs exhibited spherical or oval shapes, variations in morphology were observed due to the extraction-based synthetic procedure, leading to the detection of anisotropic forms. Poly-dispersed NPs are the stable form achieved in our work, as we exclusively employed the most practicable reducing and capping agent, starch.

Biomedical applications of nanocomposites critically depend on their cytotoxic effect on different human cells [17, 71]. In the current study, a clear low anti-proliferative outcomes of different biosynthesized St/Ag-Se NC concentrations against two malignant cell lines were reported. The least viability percentages of both cell lines were observed when exposed to the nanocomposite at 1 mg/ml. A higher value of IC_50_ (21.37 µg/ml) of St/Ag-Se NC against malignant colon cell line HCT-116, in comparing with doxorubicin IC_50_ (7.98 µg/ml), was obtained which reflect slightly low toxic activity. While, a little higher IC_50_ (19.98 µg/ml) than imatinib (18.32 µg/ml) was recorded against breast cancer cell line MCF-7. In a relatively related study, Abdallah et al. [72] recorded about 1.118 and 0.119 mg/ml as an IC_50_ for biosynthesized polyvinyl alcohol/silver (PVA/Ag) and chitosan/silver (CS/Ag) nanocomposites, respectively, against Huh-7 cell line. On the same way of our results, [73] and [74] revealed chitosan nanocomposite with LC_50_ of 0.125 mg/ml and 0.518 mg/ml against HeLa cell lines and brine shrimp, respectively. These high IC_50_ values indicated the high biosafety of the biogenic nanocomposites against the tested cell lines.

Interestingly, the low absorbance, which represents the lowest level of hemolysis, was observed at a concentration of 0.25 mg/ml of St/Ag-Se NCs. Our observations were in the same context with the study conducted by Jayeoye et al. [75] who reported an increased hemolysis percentage as the poly (vinyl alcohol-co-ethylene glycol)/poly (3-aminophenyl boronic acid) nanocomposite concentration increase. Similarly, the polypyrrole nanocomposites concentration below 2.5 mg/ml exhibited hemolysis level lower than 5% [76]. These results indicates the great influence of nanocomposites hemolytic activity by their concentration and particularly high concentration had the most significant impact on RBCs destruction compared to other tested concentrations. The enhanced nanocomposites haemocompatibility may be associated with the biocompatible character of starch, poly (vinyl alcohol-co-ethylene glycol)/poly (3-aminophenyl boronic acid) and polypyrrole. Moreover, the nanocomposites erythrocytes toxicity could be correlated to pits development on RBCs membrane with subsequent pores formation and osmotic rupture.

Our findings provided confirmation of the enhanced H_2_O_2_ scavenging activity exhibited by the biosynthesized St/Ag-Se NC. As the concentration of the nanocomposite increased, its H_2_O_2_ scavenging activity increased. When compared to the positive control, ascorbic acid (50 µg/ml), the St/Ag-Se NC (2 mg/ml) demonstrated high scavenging activity (42.84%). A related results were obtained by Hasanin et al. [43] study which recognized forceful antioxidant effect of mycosynthesized starch/copper nanocomposite, at different concentrations, with relatively equal activity to ascorbic acid. In the same context, high H_2_O_2_ scavenging percentage by Ag NPs was spectrophotometrically observed by M et al. [77] study, in comparing with ascorbic acid. At 0.1 mg/ml of Ag NPs, significant H_2_O_2_ scavenging activity (86.47%) was found. These results impressively suggest the utilization of biosynthesized nanocomposites as antioxidant agents for controlling of various degenerative disorders caused by reactive oxygen species.

In our research, the cytoplasmic potassium leakage assay illustrate the effect of biosynthesized St/Ag-Se NC on both bacterial and fungal cells membrane. In a related study, the amount of cellular protein released from bacterial cells was directly proportional to the applied nanocomposites concentration [78]. These findings clarify the cell membrane dysfunction activity of St/Ag-Se NC as a possible mechanism of nanocomposites microbial cells killing effect.

In the present study, agar well diffusion assay showed the good antimicrobial activity of St/Ag-Se NC (2 mg/ml) against all the tested microbial standard strains except *S. typhi* and *C. herbarum*. *Staph aureus, E. coli*, and *S. epidermidis* were highly affected with IZDs of 21, 21, and 19 mm, respectively in comparing with chloramphenicol (30 µg/ml) that showed IZDs 28, 24, and 24 mm, respectively. On the other hand, *C. albicans* was vastly affected with IZD 28 mm, in comparing with 32 mm IZD demonstrated with fluconazole (50 µg/ml). Our results were highly compatible with Abdallah et al. [72] who biosynthesized PVA/Ag and CS/Ag nanocomposites with clear effect on MDR bacteria. The PVA/Ag and CS/Ag nanocomposites showed significant antimicrobial activities against both Gram-positive and Gram-negative bacteria with IZDs ranging between 33 ± 3 and 21 ± 1 mm for PVA/Ag and 14 ± 2 and 8 ± 0.5 mm in case of CS/Ag. As well, their maximum activity demonstrated against *S. epidermidis* while, minimum activity was noticed against *K. pneumonia* and *S. aureus*. These inhibitory effects could be clarify the enhanced diffusion and incessant release of metal NPs from the biosynthesized nanocomposites into the nearby agar medium and subsequently large IZDs. Also, the broad range of the biosynthesized St/Ag-Se NC activity against the majority of the tested bacterial and fungal strains indicate their advantage as a non-specific antimicrobial agent.

In our study, *S. epidermidis* was the uppermost affected bacterial strains with St/Ag-Se NC MIC of 18.75 µg/ml. Also, *B. subtilis*, *S. aureus*, and *E. coli* were highly affected with equal MIC and MBC (25 µg/ml) for all strains. As well, the highest fungal destructive effect was demonstrated against *C. albicans* with MIC of 50 µg/ml. On top, *S. aureus*, *B. subtilis*, *E. coli*, *P. mirabilis*, *C. tropicalis*, and *(A) fumigatus* showed microbial tolerance ≤ 2. In related studies, lower MIC (15 µg/ml) of selenium/silver nanocomposite against *(B) subtilis* and *E. coli* was reported [79]. While, Abd-Elraoof et al. [80] quantitatively assessed chitosan/selenium nanocomposite activity against *S. typhimurium*, *S. aureus*, and *E. coli* revealing MIC of 17.5, 20, and 25 µg/ml, respectively. Additionally, our results were consistent with Amr et al. [45] findings that demonstrated biosynthesized Ag NPs with an equal MICs and MBCs (1000 µg/ml) against *P. aeruginosa*, *E. coli*, and *K. quasipneumoniae* and subsequent MBC/MIC ratio equal one. Similarly, *E. coli*, *K. pneumoniae*, *P. aeruginosa*, *Salmonella enterica*, and *Salmonella infantis* showed biogenic Ag NPs MBC/MIC ratio ≤ 2 [81]. The fungal level, selenium/chitosan nanocomposite showed activity against *(C) albicans* with low MICs of 25 µg/ml [42]. As well, Arsène et al. [82] use *Aloe vera* extract for Ag NPs synthesis with high activity against four *Candida albicans* strains where the MICs range 16–32 µg/ml. While, Chatterjee et al. [83] silver NPs prevented the tested *Aspergillus* spp. observable growth (MIC) at 8–128 µg/ml and exhibited fungicidal effect at 32–256 µg/ml. The variability of the obtained MIC values might be reflect the reducing agents variation in different biogenic method with consequential inconsistence NPs shape, size, and stabilizing agents [84–86]. Also, significant dissimilarity in microbial cells wall composition might be attributed to uneven MIC. The results also indicate the acceptable activity with low microbial tolerability of the tested strains to the biosynthesized St/Ag-Se NC and their activity as germicidal agent against the majority of tested strains. The ultrafine St/Ag-Se NC (22.6-89.45 nm) could be reflect their high efficacy at little concentration.

In the current study, the impact of different concentrations of St/Ag-Se NC on various bacterial and fungal biofilms was evident. Significant alterations in biofilm formation were observed at a concentration of 1 mg/ml. A substantial decrease of 87.5% and 82.5% in biofilm formation for *S. epidermidis* and *B. subtilis*, respectively were recorded. Furthermore, *S. aureus*, *E. coli*, and *P. mirabilis* exhibited notable reductions in biofilm formation of 68.05%, 66.12%, and 65.37%, respectively when treated with 0.5 mg/ml of St/Ag-Se NC. Our findings were consistent with Bellisario et al. [87] who recorded variable silver/polypropylene nanocomposites antibiofilm activity against *K. pneumoniae* and *S. aureus*. Additionally, El-Behery et al. [88] reported poor biofilm formation by *S. aureus*, *E. coli*, and *C. albicans* (90.62%, 90.70%, and 90.88%, respectively) after treatment with silver/selenium nanocomposite. The collected results illustrate the increased activity of bimetalic combination as well as, the proportional correlation between the nanocomposites concentration and their biofilm inhibitory effect that could be recommend their future relevance as one of the preventive measures for microbial biofilm formation.

## Conclusion

In the current study, a nanocomposite based on starch, silver and selenium was biosynthesized using ecofriendly method. The whole characters of biosynthesized St/Ag-Se Nc were determined following the UV-Vis, XRD, DLS, Zeta potential, SEM, and HR-TEM analysis. The obtained nanocomposite was pure, crystalline, oval and spherical in shape, with a mean particle diameter of 67.87 nm. The nanocomposite displayed promising antimicrobial activities against wide range of bacterial and fungal species which suggest its feasible using as an alternative broad spectrum antimicrobial agents. Their effective microbial biofilm suppression as well as, low cytotoxic and hemolytic activities indicated their possible application in biomedical intentions.

## Data Availability

No datasets were generated or analysed during the current study.
